# Modified Compression Test of Corrugated Board Fruit Tray: Numerical Modeling and Global Sensitivity Analysis

**DOI:** 10.3390/ma16031121

**Published:** 2023-01-28

**Authors:** Tomasz Garbowski, Damian Mrówczyński, Jakub Krzysztof Grabski

**Affiliations:** 1 Department of Biosystems Engineering, Poznan University of Life Sciences, Wojska Polskiego 50, 60-627 Poznań, Poland; 2 Doctoral School, Department of Biosystems Engineering, Poznan University of Life Sciences, Wojska Polskiego 28, 60-637 Poznań, Poland; 3 Institute of Applied Mechanics, Poznan University of Technology, Jana Pawła II 24, 60-965 Poznań, Poland

**Keywords:** corrugated board, fruit and vegetable tray, top-to-bottom box compression test, sensitivity analysis, finite element method

## Abstract

This article presents a modified configuration of the box compression test (BCT), which reflects the actual behavior of the vegetable or fruit trays during transport and storage. In traditional load capacity tests, trays are treated as classic transport boxes, i.e., they are compressed between two rigid plates, which does not take into account the specific geometry of this type of packaging. Both the boundary conditions and the loads acting on the tray were modified. The paper presents the concept of a new test, as well as numerical models and a sensitivity analysis of the modified BCT to the basic geometrical dimensions of the tray. The conducted research clearly shows that the proposed configuration of the load-bearing capacity test of a tray is closer to the actual operation of the packaging. As a result, most of the parameters that are not active under the conditions of the classical BCT become more important in the new configuration, which corresponds to the observations on the real performance of the packaging.

## 1. Introduction

Every year, a huge number of fruits and vegetables, such as bananas, apples, lemons, tomatoes, and peppers, are transported long distances. For example, in 2021, 21.5 million tons of bananas were transported globally. The vast majority of them (about 16.5 million tons) came from Africa. They were mainly exported to Europe and the United States [1]. This type of long-term and -distance transportation requires appropriate packaging. Open-top cartons made from corrugated board are very common in such long-distance transportation of products, including fruits and vegetables, because of many factors. First of all, such packaging is perfectly suited for transportation in environments with variations in conditions such as temperature and humidity. The main feature that ensures the cartons’ appropriate conditions is the presence of ventilation holes. However, while the presence of holes in open-top packaging ensures suitable environmental conditions, on the other hand they weaken its mechanical strength. Sing et al. experimentally proved that the loss of compression strength in packaging with hand or ventilation holes can range from 10% to 40% [2]. Furthermore, corrugated board fruit trays can be considered as environmentally friendly. Many studies show that nowadays people are paying more and more attention to eco-friendly packaging [3,4,5].

The strength of boxes made from corrugated board can be tested physically by performing some specific tests. From a practical point of view, one can distinguish the compressive, tensile, and bursting strength tests. In the packing industry, commonly used tests are the box compression test (BCT) and the edge crush test (ECT).

One can notice that conducting physical experiments is a time-, and very often also cost-consuming task. An alternative for this is performing numerical simulations. Over the years, many authors have used numerical methods to study the physical properties of corrugated cardboard, and boxes made from it. In the literature, one can find many works, in which, e.g., the buckling [6] or transverse shear [7,8] phenomena of corrugated boards have been studied. Numerical studies can also be successfully performed for corrugated board boxes with holes, examining various physical features of them. Therefore, many different numerical modelling and simulation techniques can be applied for this purpose, taking into account the physical principles of the real tray’s behavior. However, the proper modeling of the physical phenomena, i.e., a mathematical description of the problem including determination of the governing equations, loads, and boundary conditions, is not a trivial task and requires some experience in this area, in particular when complex structures and physical situations are taken into consideration.

One of the main important features of corrugated board fruit trays are the ventilation conditions. In the literature, one can find many papers in which this property of the boxes is studied using Computational Fluid Dynamics (CFD) techniques [9]. Recently, Nanga et al. investigated the transient airflow and heat transfer by convection and conduction inside a cold chamber with boxes filled with fruit [10]. The authors used a transient three-dimensional CFD model. Similarly, Ilangovan et al. studied the influence of holes’ design, including their size, shape, and position, on the ventilation features of fruit packaging boxes [11].

The strength of packaging with holes has also been examined numerically in the literature [12,13,14,15,16]. The effect of various geometrical features of the holes, including their height, area, orientation, shape, and number, on the final strength of the box was investigated by Fadiji et al. [17]. They compared numerical and experimental results. The same authors examined later the stability and strength of boxes with holes using the finite element method (FEM) [18]. Garbowski et al. proposed a numerical-analytical approach for prediction of the box compression strength in the BCT, taking into account the presence of holes [19], perforations [20], and shifted flaps [21], based on a modified well-known McKee formula [22].

Corrugated board fruit trays have a distinct construction in comparison to typical flap packaging, e.g., F0201 in keeping with FEFCO codes [14]. The structure of them is quite complex. Therefore, many analytical formulas, which give relatively good results for simple flap packaging [22,23,24,25,26,27,28], fail in the case of corrugated board fruit trays. Very often it is very difficult, or even impossible, to determine the strength of open-top packaging using analytical methods, due to the complicated geometry and difficulties with derivation of the formulas in such a case, and parameterization of the geometry. Assessing the strength of corrugated board fruit trays is not a trivial task. In industrial practice, it is possible to rely on the many years of experience of corrugated packaging design staff. Nevertheless, to avoid a qualitative assessment of the strength, computer methods should be used to quantify it. In such a situation, it is convenient to use numerical methods. However, as previously mentioned, numerical simulations need an appropriate building of the computational model, including geometry, loads, and boundary conditions, which reflect the physical phenomena as accurately as possible. This also requires experience in this area. Another approach is the application of artificial neural networks (ANNs), which after appropriate data selection and preparation, and a training process, can efficiently predict selected properties of the corrugated board and packaging made from it. In the literature, such an approach has recently been very popular. Adamopoulos et al. applied multiple linear regression and ANNs for estimation of the corrugated base paper’s properties [29]. Chaveesuk et al. predicted the ECT values using ANN [30]. Archaviboony et al. [31] analyzed the influence of hand and ventilation holes’ geometrical features on the BCT results using ANNs. ANNs have a wide area of applications [32,33,34,35], including the food and packaging industry [36,37]. They can also be efficiently used as a tool for sensitivity analysis [38], as performed in this paper.

Vegetable and fruit trays [39] made of corrugated board, are packaging characterized by a complex structure, and often travel in extreme transport conditions. Therefore, their design requires them to be stable, both in terms of static strength, and against the influence of humidity, temperature, or long-term load causing material creep. Unfortunately, in laboratories there is no developed standard for proper testing of their load capacity, even under simple static load conditions. In turn, traditional transport packaging, commonly known as flap boxes, is tested in a compression press between two rigid plates according to a well-developed standard, i.e., the box compression test (BCT). This article proposes a modification of the popular box compression test for fruit and vegetable trays. The modified approach enables a more accurate prediction of the packaging’s strength, including loads, boundary conditions, and box wall connections. The results are used in the FEM model, and they reflect the actual behavior of the packaging. In order to verify the correctness of the proposed BCT modification, a sensitivity analysis of the FEM model of the packaging to small changes in the basic geometric parameters was also carried out. The work proves that some geometric features of the tray, inactive in the classic BCT tests, become more important in the modified test. Therefore, the tray design process should take these changes into account and be updated with these observations.

## 2. Materials and Methods

### 2.1. Global Sensitivity Calculations

The best tool to check the impact of each component on the performance of the entire system is a sensitivity analysis. However, checking the effect of each parameter at one specific point in the parameter space does not give a complete picture of the model’s sensitivity. In order to examine the overall effect of the parameters on the model’s performance in the full range of variability of each parameter, a global sensitivity analysis is usually adopted. It is a global sensitivity analysis in the sense that the simulations are performed at different locations in the parameter space, and not just at one local point. This provides information not only about the local relationship between the parameter and the starting point, but also about the global significance of each parameter. This kind of sensitivity calculation approach was also used in [40,41].

In this study, a sensitivity analysis of the modified BCT was performed for 20 packaging geometries (which represent the 20 different locations in the parameter space) and three types of cardboard. A discussion on the selection of these points will be presented in the next section. Selected geometric features of the packaging, their designation and specific starting values of these parameters, will also be discussed in the next section. For each geometry, a set of parameters was collected as a vector x, and the value of the load-bearing capacity, denoted as $h\left(x\right)$, was calculated for this set of box dimensions. In the next step, the subsequent i-th parameters were perturbed with small values of $\Delta x_{i}$ to determine the local change of the investigated quantity $h\left(x\pm e_{i}\Delta x_{i}\right)$, where $e_{i}$ is the unit vector of the i-th parameter in the explored space. The sensitivity of the compressive strength to a change in the *i*-th parameter can be calculated by the following formula:

(1) $$s=\frac{h\left(x+e_{i}{\Delta x}_{i}\right)-h\left(x-e_{i}\Delta x_{i}\right)}{2\Delta x_{i}}\frac{x_{i}}{h\left(x\right)}\;.$$

### 2.2. Parametric Model of Open-Top Cartons

**Figure 1:**
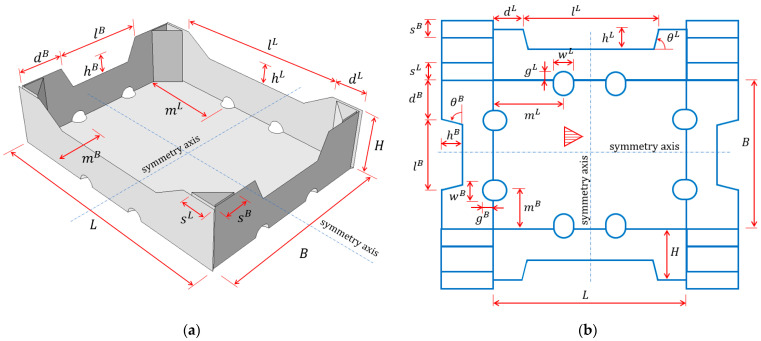
Parametric model of the open-top tray: (**a**) 3D view and (**b**) 2D scheme (for clarity, $w^{L}$, $g^{L}$, $w^{B}$, $g^{B}$, $\theta^{L}$, and $\theta^{B}$ parameters are not shown in the first drawing).

There are many types of fruit and vegetable trays on the market, depending on the specific needs and transport requirements. Despite the differences, there are some similarities, such as repetitive folds and packaging design. Manufacturers also offer their own designs, which were created based on experience, and often intuition, rather than solid analysis. A type of tray that is quite common on the packaging market was adopted for this study. Figure 1 shows the geometry of the selected tray. In these cartons, the sidewalls fold vertically and the rectangular panels form stiffening triangles. The rigidity of the corner is ensured by gluing one of the stiffening triangle walls to the longer sidewalls. Sometimes there is also a trapezoidal fold on each sidewall, which is bent down and glued to stiffen the sidewall (not shown in the drawings).

On the sidewalls and the bottom of the box, there are round or oval ventilation holes, which ensure free flow of air. In the classic BCT laboratory test, these holes have no real effect on the load capacity of the package, which does not coincide with the observations of the real product operation. It was this discrepancy between the measured and estimated influence of geometric features on the load capacity of the packaging that was the main motivation for conducting these studies.

Seventeen design parameters were selected for the analysis, which can be specified in the selected type of packaging. Figure 1 shows all analyzed parameters. The choice was guided by the assumed impact of a given parameter on the load-bearing capacity and its importance for proper moisture draining and other functions of the packaging. The following geometrical parameters were selected:

- L and B—the length and width of the packaging, respectively;
- H—the height of the stiffening triangles and the carton;
- $d^{L}$ and $d^{B}$—half of the horizontal length of the non-folded part of the longer and shorter sidewalls, respectively;
- $h^{L}$ and $h^{B}$—the width and height of the trapezoidal folds on the longer and shorter sidewalls, respectively;
- $s^{L}$ and $s^{B}$—the length of the sides of the stiffening triangles on the longer and shorter sidewalls, respectively;
- $w^{L}$ and $g^{L}$—the width and height of the ventilation holes on the longer sidewalls, respectively;
- $m^{L}$—the distance of the ventilation holes on the longer sidewalls from the shorter sidewalls to its axis;
- $w^{B}$ and $g^{B}$—the width and height of the ventilation holes on the shorter sidewalls, respectively;
- $m^{B}$—the distance of the ventilation holes on the shorter sidewalls from the longer sidewalls to its axis;
- $\theta^{L}$ and $\theta^{B}$—inclination of the arms of the trapezoidal folds on the longer and shorter sidewalls, respectively.

Note that the modifications of both $d^{L}$ and $d^{B}$, as well as L and B, change the widths of the trapezoidal folds on the sidewalls $l^{L}$ and $l^{B}$, therefore, these parameters are not taken into account in the performed calculations.

The study included the analyses of many packaging geometries, therefore it was necessary to create an algorithm to automatically generate numerical models of the trays. For this purpose, an algorithm was created in the MATLAB software, which automatically generated FEM models in the form of batch files for the Abaqus FEA, involving all the geometric parameters specified above. The box compression test (BCT) of each case was then simulated. The next step, after creating an efficient numerical algorithm, was the selection of twenty sets of geometric parameters in a seventeen-dimensional parameter space. For this purpose, the Latin Hypercube Sampling (LHS) method was utilized. An example of the LHS strategy was presented by Jin et al. [42] or by Buljak and Garbowski in [43]. In Table 1, the values of all seventeen parameters for the twenty boxes’ geometries are shown. The selection of such a large number of starting points using optimized LHS, enables a very even sampling of the space of the design parameters. This makes it possible to explore the parameter space in twenty locations very well, which helps to understand the relationship between the model sensitivity and the location of each initial point. This approach allows the determination of both the global significance of each parameter and the local response in the selected points.

### 2.3. Numerical Model of Open-Top Boxes

**Figure 2:**
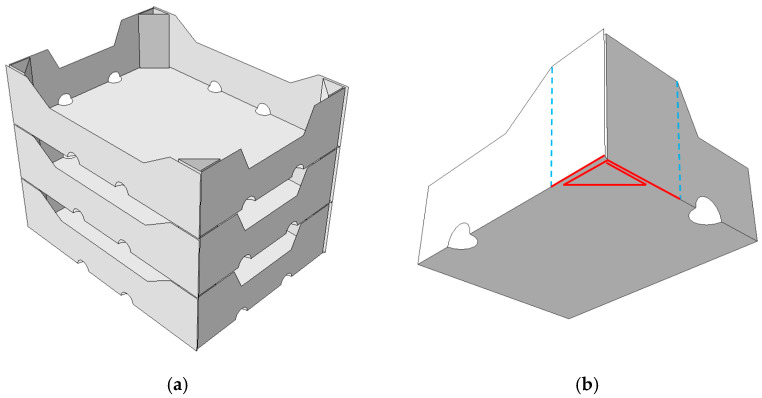
Support schemes of open-top cartons: (**a**) stacking during transport or storage, and (**b**) bottom support sections.

The main numerical computations were performed in the commercial FEM software [44] Abaqus Unified FEA (version 2021, Dassault Systemes SIMULIA Corp., Johnston, RI, USA), where a modified compression test of open-top cartons was simulated. In Figure 2a, the orientation of the boxes during transport and storage is shown. It can be clearly seen that the trays are supported on the upper edges of the trays that lie underneath them. In Figure 2b, the support sections in the 1/4 open-top carton model are marked in red. This approach is significantly different from the standard box compression test, in which the vertical displacement of the entire tray bottom is blocked. In addition, due to only partial support, the modified approach must include the load of the transported goods acting on the bottom of the box.

**Figure 3:**
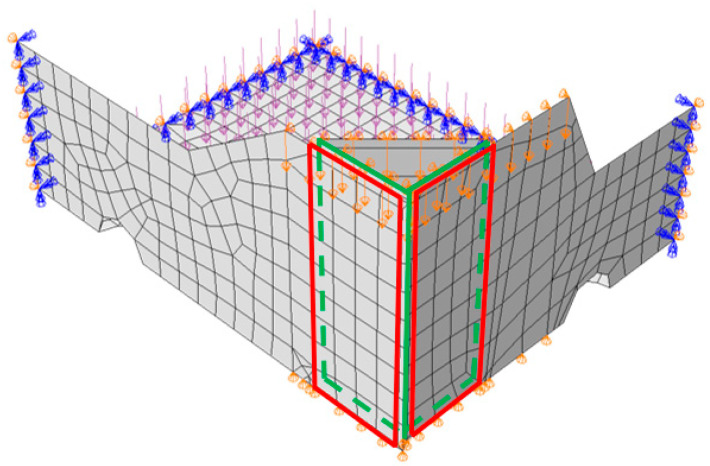
Finite element model of open-top cartons with boundary conditions and mesh (the red and green areas represent glued fragments of the panels).

The numerical models, due to their obvious double symmetry, consisted of only 1/4 of the tray. The application of this symmetry condition helped in reducing the number of finite elements, and thus sped up the computations. Such a simplification was made possible by applying the appropriate symmetrical boundary conditions on the edges of the sidewalls and the bottom of the box (see Figure 3), which ensured the correct operation of the model. In addition, the out-of-plane displacement of the top edges was also blocked.

As part of the analysis, three computation steps were carried out. The first step was the buckling analysis, in which the first buckling mode was determined and then applied as geometrical imperfections to the packaging model. In the second step, the carton was supported on the edges marked in Figure 2b and loaded with a pressure simulating a 10 kg product. In the third computational step, the compression test was simulated by applying a vertical displacement to the top edges of the box. The panels of the stiffening triangle and fragments of the side walls, which are glued together in order to assemble the packaging, are also marked in red and green colors. In the FE models, it was modeled using a technique called ‘tie’, which ensures the continuity of displacements of the two connected areas.

As already discussed, here the sensitivity analysis was made for three different corrugated boards (i.e., materials), which are described as a linear elastic orthotropic model with Hill plasticity [45]. In Table 2, the mechanical parameters of the materials used are presented. Cardboard data were determined by the BSE System via FEMAT [46] based on various laboratory tests of cardboard. To obtain reliable and representative measurement values, a minimum of ten samples, previously conditioned in a climate chamber, were tested in each test. Each cardboard had been marked with a symbol that showed the type of wave and grammage in $g/m^{2}$. The columns of Table 2 contain: corrugated cardboard grade, elastic parameters (moduli of elasticity $E_{1}$ and $E_{2}$, Poisson’s ratio $\nu_{12}$, in-plane shear stiffness $G_{12}$, and transverse shear stiffnesses $G_{13}$ and $G_{23}$), and plastic material parameters (initial yield stress $\sigma_{0}$ and yield stress ratio in the machine direction $R_{11}$).

As part of the procedure, a compression test of 20 packaging geometries (see Table 1) was simulated for the three corrugated boards described in Table 2. In order to compute the sensitivity, each of the 17 parameters were perturbed by 1%, which resulted in 18 analyses for each of the 20 packaging designs. In total, 1080 numerical BCT simulations (3 materials × 20 geometries × 18 analyses) were carried out. In each case, the model consisted of 4-node quadrilateral shell elements with full integration, named S4, and was supplemented if necessary with 3-node triangular shell elements with full integration, named S3 according to Abaqus Unified FEA. In each model, a global mesh size of 10 mm was adopted, which resulted in a different number of elements depending on the tray. For example, for the first case, 841 nodes and 774 elements were obtained (760 quadrilateral and 14 triangular elements), see Figure 3.

The arbitrarily given choice of the size of the finite elements is based on the experience presented in previous works, where a thorough analysis of the influence of the mesh size on the results of the numerical analyses of various trays has already been carried out and presented [38].

## 3. Results

The modification of the support condition in the BCT made it necessary to add a load on the bottom of the open-top carton. Therefore, before the main calculations of the load capacity of the tray, the influence of the pressure exerted by the fruit or vegetables on the load-bearing capacity of the tray was checked. For this purpose, the compressive strength of the first geometry case was analyzed for three load values and compared with the load capacity of the tray without any load on the bottom. In Table 3, the BCT decreases for the analyzed load values are presented. The obtained results show that the effect of the pressure exerted on the bottom is rather negligible, therefore it was not parameterized in further analyses and was fixed as a constant value of 10 kg. It is worth noting that the load applied to the bottom of the tray is not a load causing damage to the box, and only slightly reduces the load capacity of the tray.

**Figure 4:**
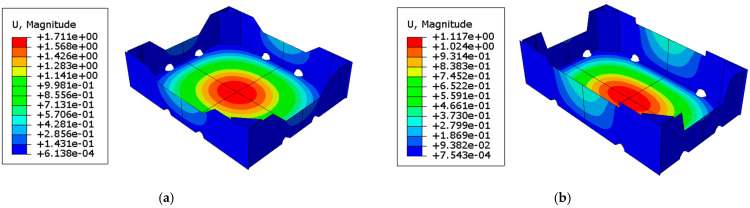
Selected displacement maps after two computational steps: (**a**) case 19 and (**b**) case 20.

As already described in the previous section, at the beginning the buckling analysis was performed, from which the displacement distribution of each open-top carton panel was obtained. Then, the displacement maps were applied as initial imperfections and the bottom of the box was loaded with pressure simulating the stored goods. Figure 4 shows the displacements after the second computational step for two selected cases.

**Figure 5:**
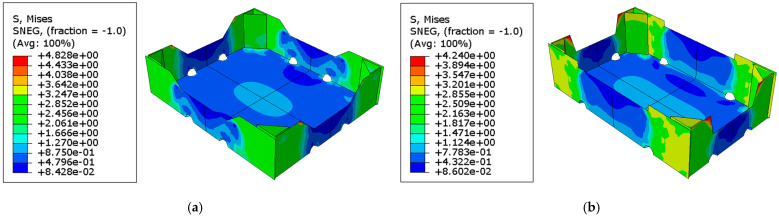
Distribution of effective Huber–Mises–Hencky stresses at the moment of reaching the maximum strength of the fruit tray: (**a**) case 19 and (**b**) case 20.

In the last step, a vertical displacement of the top edges was applied, which simulated a modified box compression test. This allows the construction of a force-displacement diagram and thus the packaging strength. In Figure 5, distributions of the Huber–Mises-–Hencky effective stresses for two representative geometry cases, 19 and 20, are shown.

As described in Section 2, a sensitivity analysis was performed for 20 packaging geometries and three types of cardboard. Considering each variant of material and geometry, the reference strength of the open-top carton was first determined. Then, the load-bearing capacity was computed for the same set of parameters, with one parameter perturbed by 1%. As a result, a total of 1080 box strength values were obtained, which allowed the determination of the sensitivity of the modified BCT to the change of 17 geometrical parameters at 20 starting points for three different cardboards. All sensitivity values were calculated according to Equation (1), where vector x contains the geometric parameters and $h\left(x\right)$ is the packaging strength for a certain set of parameters. Table 4 shows the sensitivities for selected EB-965 corrugated boards.

It is worth noting (in Figure 5) the stress distribution in both cases in the context of the location of the ventilation openings. Figure 5b shows that the opening is located in the load-bearing zone of the tray corner, while the openings shown in Figure 5a are outside this zone. Their location can, therefore, affect the bearing capacity, which will be discussed in the next section.

**Figure 6:**
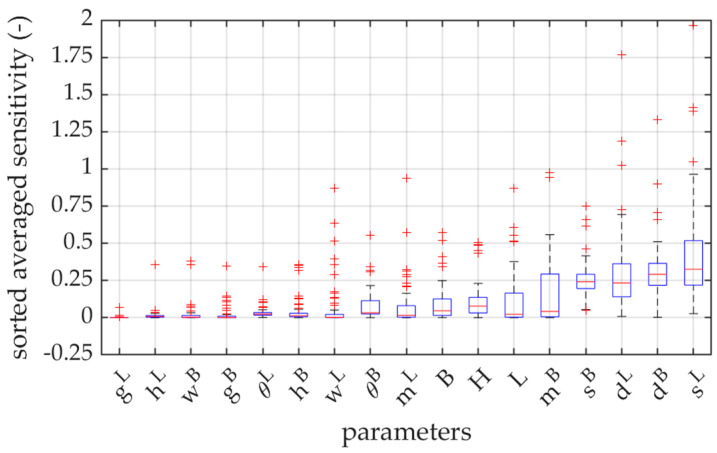
Sensitivity box plots of the 17 parameters considered (sorted in ascending order).

To compare the influence of the parameters on the open-top carton strength, the average sensitivity of each parameter from 20 geometries was calculated. The median of the computed sensitivities is also marked with red dots. Figure 6 shows box plots of sensitivity to each parameter for all three types of corrugated board. In the graph, the bottom and top of each box are the 25th and 75th percentiles of the sample, respectively. The distance between the bottom and top of each box is the interquartile range. The red line in the middle of each box is the sample median. If the median is not centered in the box, the plot shows sample skewness. The whiskers are lines extending above and below each box. Whiskers go from the end of the interquartile range to the furthest observation within the whisker length (the adjacent value). Observations beyond the whisker length are marked with red + as outliers.

**Figure 7:**
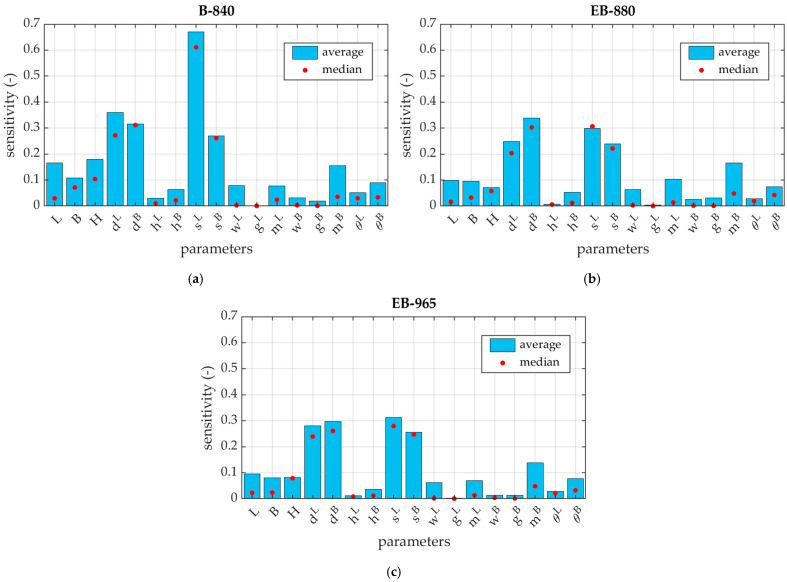
Average and median sensitivities for 17 analyzed parameters for three different cardboards: (**a**) B-840, (**b**) EB-880, and (**c**) EB-965.

In addition, in Figure 7, the average sensitivities for the three cardboards are presented as bar graphs.

## 4. Discussion

All the crucial results have been shown in Figure 4, Figure 5, Figure 6 and Figure 7 and Table 3 and Table 4. Before the main calculations, a study of the load on the bottom of the packaging was carried out, which consisted in checking the impact of the applied pressure simulating loads coming from the transported fruit or vegetables on the final packaging strength. In Table 3, the load capacity reductions are shown depending on the applied weight. It can be seen that increasing the load does not reduce the strength of the tray. This means that both the deformation of the load-bearing walls of the packaging due to the bottom load, and the deformation of the bottom itself, have no effect on the load-carrying capacity. This observation leads to the conclusion that the trays have a much higher bottom loading capacity than the stack loading capacity.

In Figure 4, displacement maps are shown after a buckling analysis and applying pressure to the bottom of the open-top carton. It can be seen that, as expected, the maximum displacements occur at the box bottom in all cases. The maximum displacements on the side walls are obtained on the longer sidewalls, which is due to the greater slenderness of these panels. However, these deformations do not weaken the load capacity of the box.

Figure 5 shows the Huber–Mises–Hencky effective stresses when the tray reaches its maximum strength. It may be observed that the maximum effective stresses occur on the sidewalls at the packaging corners. All presented cases show the highest values of effective stresses in the corner zones as expected. Due to the modified support conditions in the box compression test, in the middle of the sidewalls’ span, the effective stresses are very small. This effect can be well captured in comparison to the work of Mrówczyński et al. [38], where the standard box compression test was carried out. Due to the full support of the bottom in the standard test, high effective stress values are also reached beyond the corner zones.

In this work, the sensitivities of the modified BCT were calculated for three corrugated boards, named B-840, EB-880, and EB-965. Due to the similarity of the results, the sensitivity values are only shown for EB-965 grade in Table 4. Figure 6 and Figure 7 show the average sensitivities of the 17 analyzed parameters for the three materials using bar, and box and whisker, plots. The obtained results show that the parameters $d^{L}$, $d^{B}$, $s^{L}$, and $s^{B}$, which are the dimensions of the top edges of the packaging, have the highest impact on the box strength. This means that only the corners of the tray actually work in the box compression test. The method of support, completely different than in the case of the traditional BCT test, enhances this effect even more. Ultimately, some kind of compressed columns of material are formed in each corner, that carry all the load acting on the tray.

It appears that the location of the ventilation holes on the sidewalls and the bottom $m^{L}$ and $m^{B}$ also have a great importance. The dimensions of the ventilation holes $g^{L}$, $w^{L}$, $g^{B}$, and $w^{B}$, the height of the trapezoidal folds $h^{L}$ and $h^{B}$, and the inclination of the arms of these trapezoidal folds $\theta^{L}$ and $\theta^{B}$, do not have a significant influence on the load-bearing capacity. The above observations lead to very clear conclusions regarding the impact of the ventilation holes on the load capacity of the entire package. As already noted, the load bearing part of the tray is concentrated in the corners, therefore the effect of the holes is noticeable only when their locations are in these load bearing areas of the tray.

If one looks at the results for all geometries separately, it can be seen that for some parameters the sensitivity of the model is very high, while the average value remains low. This situation occurs mainly for the parameters L, B, $h^{L}$, $h^{B}$, $w^{L}$, $m^{L}$, $w^{B}$, and $m^{B}$, when the perturbation of these parameters interferes with the load-bearing zone. In Figure 6, it can be clearly seen that in many cases the average value (or median) of the model’s sensitivity to individual parameters is close to zero, and some outliers oscillate around 1 or 2. This means that one should be very careful in the process of designing the tray, because some combinations of geometrical parameters that usually do not have a great impact on the performance of the package, can play a key or at least a significant role.

The sensitivity of the parameters in the modified box compression test can be compared to the results obtained from the standard approach [38]. The top edge lengths $d^{L}$, $d^{B}$, $s^{L}$, and $s^{B}$ are the most significant in both methods, because the wider the load-bearing panels are, the more material is involved in the bearing zone of each corner. In the modified approach, trapezoidal folds have less influence, which results from the change in the support conditions. The location of the ventilation holes on the sidewalls gains in importance in the modified test, because decreasing their distance from the corner zones can reduce the support sections of the packaging.

## 5. Conclusions

This paper presents an extended sensitivity analysis of the model for estimating the compressive capacity of a vegetable or fruit tray in the modified box compression test. The proposed modification mainly concerns the change of the boundary conditions in the model, so as to bring the nature of the numerical simulation closer to the actual operation of the packaging during storage and transport. In the modified test, the sensitivity of the model to some geometrical parameters of the packaging clearly changes in relation to the sensitivity of the model to the geometrical parameters of the same packaging in the classical BCT test. This gives a very clear signal for tray designers to focus on slightly different parameters of the box than they have to date.

From the analyses carried out, a basic conclusion can be drawn that in the modified BCT test only the parameters describing the geometry of the corners have a significant impact on the load capacity of the packaging. This also applies to the traditional BCT—the difference appears in the sensitivity of the tray’s load capacity to the location of the ventilation holes. It was discovered in this study, that the influence of the location of the ventilation holes on the load capacity of the tray is also important in the new configuration of the BCT—however, it is noticeable only when the holes are placed in the working zone of the corner. The geometry of the holes themselves does not significantly affect the load capacity of the corners, even when the presence of the holes directly weakens the load capacity of the corners.

## Figures and Tables

**Table 1:** Dimensions of selected packaging geometries; all parameter values, except the last two columns, are presented in mm.

Box Case	L	B	H	$d^{L}$	$d^{B}$	$h^{L}$	$h^{B}$	$s^{L}$	$s^{B}$	$w^{L}$	$g^{L}$	$m^{L}$	$w^{B}$	$g^{B}$	$m^{B}$	$\theta^{L}$ $\left({}^{\circ}\right)$	$\theta^{B}$ $\left({}^{\circ}\right)$
1	366	282	94	46	70.5	30	37	36	31.5	25	12.5	134.5	25	12.5	91.5	33.5	65
2	330	282	94	46	70.5	30	37	36	31.5	25	12.5	134.5	30	15	91.5	33.5	65
3	366	254	94	46	70.5	30	37	36	31.5	30	15	134.5	25	12.5	91.5	33.5	65
4	366	282	85	46	70.5	30	37	36	31.5	25	12.5	90	25	12.5	70	33.5	65
5	366	282	94	76	70.5	30	37	36	31.5	20	10	114.5	20	10	111.5	90	90
6	366	282	94	73	70.5	30	37	50	40	32	16	134.5	36	18	91.5	33.5	65
7	386	282	94	83	70.5	30	37	30	40	32	16	134.5	25	12.5	91.5	45	45
8	386	302	94	83	80.5	40	40	30	40	32	16	134.5	25	12.5	91.5	75	60
9	391	262	104	85.5	60.5	40	40	25	25	28	14	125	25	12.5	91.5	75	60
11	396	262	90	88	60.5	40	40	45	32	35	14	125	30	12.5	91.5	80	85
12	401	265	98	80.5	52	20	20	38	40	34	17	125	28	14	91.5	55	45
13	386	268	98	100	83.5	20	20	38	32	20	10	125	20	10	91.5	55	45
14	388	271	98	91	75	15	25	42	45	20	10	155	20	10	111	55	45
15	392	252	94	93	65.5	15	25	30	27	20	10	92	20	10	85	55	45
16	396	252	81	48	41	15	25	30	27	20	10	135	20	10	111	20	35
17	398	252	81	49	41	35	35	30	27	20	10	135	20	10	111	37	35
18	396	252	83	67.5	51	25	35	36	36	35	10	135	35	10	85	65	55
19	310	290	85	68	70	25	35	30	28	16	8	95	16	8	85	65	55
20	320	271	81	73	55.5	25	35	40	28	22	11	115	22	11	95	40	45

**Table 2:** Corrugated board data used in the constitutive models.

Grade	$E_{1}$	$E_{2}$	$\nu_{12}$	$G_{12}$	$G_{13}$	$G_{23}$	$\sigma_{0}$	$R_{11}$
	(MPa)	(MPa)	(–)	(MPa)	(MPa)	(MPa)	(MPa)	(–)
B-840	2032	1111	0.40	1184	7	11	3.05	0.95
EB-880	1636	907	0.40	963	8	11	3.50	0.65
EB-965	1616	750	0.44	898	7	11	3.01	0.74

**Table 3:** Change in the BCT parameter (compressive strength) as a result of increasing the bottom load.

Load	ΔBCT
(kg)	(%)
5	−0.30
10	−0.57
15	−1.05

**Table 4:** Sensitivities calculated for EB-965 cardboard with min/max values marked in red and green, respectively, for all considered parameters (the darker color the more important parameter).

Case	L	B	H	$d^{L}$	$d^{B}$	$h^{L}$	$h^{B}$	$s^{L}$	$s^{B}$	$w^{L}$	$g^{L}$	$m^{L}$	$w^{B}$	$g^{B}$	$m^{B}$	$\theta^{L}$	$\theta^{B}$
1	−0.01	−0.01	−0.02	0.53	0.90	0	0	0.17	0.66	−0.01	0	0.57	0	0	0.56	0.01	0.03
2	0.01	−0.02	0.08	−0.01	0.34	0.01	0.01	0.41	0.25	0	0	0	0	0	0	0.02	0.03
3	0	0	0.01	−0.03	0.36	0.01	0.01	0.41	0.25	0	0	0.01	0	0.01	0.01	0.02	0.03
4	0	−0.01	0.11	−0.06	0.02	0.01	−0.02	0.20	0.27	0	0	0.01	−0.04	−0.02	0.29	0.03	0
5	−0.02	−0.02	0.17	0.52	0.66	0.02	−0.02	0.43	0.24	0	0	0	0	0	0	0.11	0.22
6	−0.23	0.02	0	0.40	0.21	0.01	0	0.29	0.27	0	0	−0.02	−0.07	0	−0.10	0.02	0.02
7	0	−0.22	−0.12	0.24	0.13	0.01	−0.32	0.64	0.30	0	0	−0.01	−0.01	0.06	−0.31	0.02	−0.31
8	0.02	−0.06	0.02	0.22	0.39	0	0.02	0.31	0.28	−0.29	0	−0.05	0.01	−0.05	0.09	0.03	0.02
9	0	0.09	−0.11	0.30	0.38	0	0.02	0.20	0.22	0	0	0	0	0	0	0.04	0.10
10	−0.13	0	−0.23	0.54	0.12	0.04	−0.01	0.27	0.09	0	0	0.02	0.03	0	0.03	0.07	0.14
11	−0.52	−0.09	−0.16	0.17	0	0.01	−0.01	−0.26	0.30	−0.52	0	−0.21	0	0	−0.17	0.02	0
12	−0.10	0	−0.08	0.07	0.23	0.01	0.01	0.06	0.23	−0.14	0	−0.09	0	0	−0.35	0.02	0.02
13	0.01	−0.20	−0.07	0.22	0.29	0.02	0.01	0.10	0.11	−0.03	0	0.01	0	0	0.01	0.03	0.02
14	0.10	0.11	0.13	0.26	0.42	−0.02	0	0.22	0.20	−0.01	0	0.16	0	0	0.35	0.01	−0.03
15	0.24	−0.05	−0.04	0.36	0.23	0.02	0	0.68	0.46	0	0	0	0	−0.01	0.01	0.02	0.03
16	0.26	0.25	0.09	0.24	0.31	0.01	0.02	0.32	0.23	0	0	0.08	0.02	0	0.01	0.02	0.10
17	0	0	0.16	0.31	0.26	−0.02	0.13	0.26	0.30	0	0	0	0.02	0.11	0	0	0.12
18	−0.17	0.02	−0.03	0.17	0.16	0	−0.06	−0.49	0.06	−0.17	0.02	−0.02	0.01	0	0.41	0.02	0.15
19	0	0.41	0.01	0.73	0.25	0.01	0.01	0.49	0.20	0	−0.01	−0.01	0	0	0	0.02	0.02
20	−0.07	0.02	−0.01	0.24	0.26	0	0.05	0.03	0.19	−0.08	0	−0.12	0	0	0.06	0.02	0.15

## Data Availability

The data presented in this study are available on request from the corresponding author.
